# Levels of physical activity in people with chronic pain

**DOI:** 10.4102/sajp.v73i1.323

**Published:** 2017-03-31

**Authors:** Romy Parker, Emma Bergman, Anelisiwe Mntambo, Shannon Stubbs, Matthew Wills

**Affiliations:** 1 Department of Health and Rehabilitation Sciences, Division of Physiotherapy, University of Cape Town, South Africa; 2 Klipfontein Mitchell’s Plain Substructure Community Health Centre, South Africa; 3 Bhisho Hospital, South Africa; 4 Johannesburg Metro District Clinic, South Africa; 5 Bheki Mlangeni District Hospital, South Africa

## Abstract

**Background:**

People who suffer from chronic pain are thought to have lower levels of physical activity compared to healthy individuals. However, there is a lack of evidence concerning levels of physical activity in South Africans with chronic pain.

**Objectives:**

To compare levels of physical activity in a South African sample of people with chronic pain compared to matched controls.

**Methods:**

A cross-sectional study was conducted with 24 participants (12 with chronic pain and 12 in the control group matched for age, gender and residential area). Convenience sampling was used. The participants with chronic pain (12) were identified from the Groote Schuur Hospital, Chronic Pain Management Clinic (CPMC) waiting list and had not yet received any chronic pain management intervention. Healthy matched controls were selected from volunteers in the community. With the desired alpha level set at 0.05 and the power at 0.9, 45 participants were required to detect a minimum of a 50 per cent difference between groups in levels of physical activity as measured in steps per day using pedometers. The international physical activity questionnaire (IPAQ) and the brief pain inventory (BPI) were used as measures of physical activity and pain. Objective indicators of physical activity that were used included the 6-minute walk test (6MWT), repeated sit-to-stand test (RSST), 7 days of pedometry and body mass index (BMI).

**Results:**

The chronic pain group performed significantly worse on the 6MWT (335 m [30–430] vs 680 m [430–795]; *U* = 0.5; *p* < 0.01) and on the RSST (17.9 s [11.83–105] vs 7.85 s [5.5–11.5]; *U* = 0; *p* < 0.01). The chronic pain group also had significantly lower scores on pedometry (mean daily: 2985.1 [32.8–13785.4] vs 6409.4 [4207.1–15313.6]; *U* = 35; *p* < 0.03). The BMI for the chronic pain group was significantly higher than matched controls (29.36 kg/m^2^ [18.94–34.63] vs 22.16 kg/m^2^ [17.1–30.86]; *U* = 34; *p* < 0.03).

**Conclusion:**

Participants with chronic pain had a reduced capacity for physical activity. The pedometry results illustrate a range of maladaptive strategies adopted by those with chronic pain. The majority of people with chronic pain appear to avoid physical activity leading to greater disability as a result of immobility and muscle atrophy. However, a small subgroup appears to ignore their pain and push themselves physically despite their pain. This perseverance behaviour leads to further pain as a consequence of muscle and joint overuse. Both maladaptive behavioural responses result in further sensitisation of the central nervous system. The method used to target physical activity in these patients should be considered in treatment planning, specifically for physiotherapy.

## Introduction

Physical activity has a wide range of benefits in terms of improving general health. Further, quality of life is reported to be higher in individuals who participate in regular physical activity when compared to those with a more sedentary lifestyle (Brown et al. 2003). Physical activity also has specific benefits, preventing various severe chronic diseases and conditions including cardiovascular disease, stroke, diabetes mellitus, hypertension, obesity, osteoporosis and cancer (Warburton, Nicol & Bredin 2006). The benefits of physical activity extend to improving mental health with physical activity reducing levels of stress and tension and alleviating depression (Fletcher et al. 1996; Silvestri 1997). In addition, a number of different studies have found that physical activity alleviates pain, including chronic pain in a variety of conditions (Parker, Jelsma & Stein 2016; Rainville et al. 2004; Tse, Wan & Ho 2011). It appears that, in people with chronic pain, participating in physical activity can reduce fear of pain and improve perceptions of physical and psychological well-being (Risch et al. 1993; Tse et al. 2011; Vlaeyen & Linton 2000). The above-mentioned benefits of physical activity, can be achieved through moderate-intensity exercise performed in a non-structured manner – such as standing up and walking around from a desk-bound job (Dansie et al. 2014; Sothern et al. 1999), but are most effective if incorporated into activities of daily living to become routine such as using stairs rather than elevators (Draper, Kolbe-Alexander & Lambert 2009).

Low levels of physical activity are a global concern (Hallal et al. 2012). In the UK, only 37% of men and 25% of women meet the recommended targets for physical activity (150 min of moderate-intensity exercise per week), resulting in a contribution of £1 billion to the overall health cost burden to the UK National Health Service (Allender et al. 2007). South Africa has particularly low levels of physical activity compared to global populations, with 49% of men and 43% of women having insufficient levels of physical activity (Joubert et al. 2007), significantly worse than the global inactivity level of 31% (Hallal et al. 2012). Levels of physical activity are often lower in people with chronic health conditions such as chronic pain.

Chronic pain is pain that persists after tissue healing has occurred (Azevedo et al. 2012). Chronic pain has an impact on multiple spheres of an individual’s life such as psychological state, mood and activities of daily living (Björnsdóttir, Jónsson & Valdimarsdóttir 2013; Elliott et al. 1999; Fine 2011).

In a study conducted on 15 people with chronic low back pain (CLBP) in Glasgow, Scotland, Ryan and colleagues found that people with CLBP had lower levels of physical activity compared to their matched controls (Ryan et al. 2009). Although the issue of small sample size and lack of reporting of comorbidities in this study could lead to confounding of data, the results are supported by the results from a Turkish study on 96 people with CLBP and neck pain (Soysal, Kara & Arda 2013). The Turkish study found that people with chronic pain had significantly lower levels of physical activity (International Physical Activity Questionnaire – IPAQ) and poorer scores for sleep quality (Pittsburgh Sleep Quality Index), disability (Oswestry Disability Index, Neck Disability Index) and depression (Beck Depression Inventory). The reduced levels of physical activity in both studies support the hypothesis that people with chronic painful conditions appear to be less physically active than healthy controls and these lowered levels of physical activity may contribute to increased disability.

Although a number of international studies investigating the benefits and levels of physical activity in people with chronic pain have been conducted and summarised in a systematic review (van Weering et al. 2007), none could be found from African, and specifically South African, populations. The relevance of these international studies needs to be established in South Africa. Should the South African population with chronic pain suffer from similar low levels of physical activity as reported in other populations, they are at increased risk for developing secondary conditions. In addition, should they suffer from reduced levels of physical activity, it would indicate the need for physiotherapy management to target physical activity to minimise disability.

The aim of this study was to compare levels of physical activity in people with chronic pain referred to the Groote Schuur Hospital (GSH) Chronic Pain Management Clinic (CPMC) compared with healthy matched controls. We hypothesised that people with chronic pain would have lower levels of physical activity compared to healthy individuals.

## Research design

A cross-sectional, clinical, descriptive comparative design was used.

### Participants

Participants with chronic pain were recruited from the waiting list of the CPMC at GSH. Healthy matched controls were selected from volunteers in the community.

Inclusion criteria for both groups were males and females between the ages of 18 and 60 and willing to wear a pedometer for one week. Inclusion criteria for the chronic pain group were pain for more than 6 months and for the matched controls in stable health for 6 months and no chronic pain.

The exclusion criteria were developmental disorders, the use of assistive devices for mobilisation and the presence of a lower limb amputation.

CPMC referral letters were inspected for possible participants who were phoned for further screening. Matched controls were screened telephonically for inclusion, exclusion and individual matching criteria.

### Sample size

The sample size for the study was determined based on data from McDonough et al. (2013) measuring walking using pedometers in people with CLBP. With the desired alpha level set at 0.05 and the power at 0.9, 45 participants were required to detect a minimum of a 50 per cent difference between groups in levels of physical activity as measured in steps per day using pedometers (McDonough et al. 2013). The final sample size was smaller than the desired size because of a limited number of participants available during the short time available for the study to be completed. The median age of the chronic pain group was 43 (19–54) and the median age of the control group was 47 (20–56) years.

### Measurement instruments

All measurement instruments were available in English, Afrikaans and isiXhosa and were chosen based on their validity and reliability. The following instruments were used in this study.

#### Brief pain inventory

The brief pain inventory (BPI) is a widely used and well-validated questionnaire which assesses pain severity and interference with daily functioning (Cleeland & Ryan 1994). The questionnaire includes 11 questions which generate a pain severity score (PSS) and a pain interference score (PIS) and has been validated for use in several South African languages (Parker, Jelsma & Stein 2015). Categorical variables were used to group the participants into either the chronic pain group or no chronic pain group and to eliminate any participants who did not fall discretely into these specific groups.

#### International physical activity questionnaire (short version)

The IPAQ was developed by a group of experts in 1998 as an instrument for surveillance of levels of physical activity on a global level (Lee et al. 2011). Since then, it has become the most widely used questionnaire to assess physical activity levels showing reliability and validity internationally for patients between the ages of 18 and 65 (Mestek, Plaisance & Grandjean 2008). Categorical variables were used to group the participants into low, moderate or high physical activity level based on their IPAQ scores.

#### 6-minute walk test

The 6-minute walk test (6MWT) is a commonly used measurement tool with good reliability and validity (Montgomery & Gardner 1998; Rikli & Jones 1998). This test is an objective measure of activity tolerance and physical performance measuring total walking distance covered in 6 min (Du et al. 2009). The interclass correlation coefficient of repeated 6MWT ranges from 0.75 to 0.97, indicating an adequate to excellent reliability. In a systematic review, Du and colleagues found the correlation between the 6MWT distance and the Short Form-36 physical functional domain to be 0.623 (*p* > 0.001), suggesting moderate validity of the measure.

#### Timed repeated sit-to-stand test

The repeated sit-to-stand test (RSST) has been used for a number of purposes, including as a measure of physical performance (Tsunoda et al. 2013), predicting levels of disability (Netz, Axelrad & Argov 2007), and assessing functional ability of people with CLBP (Smeets et al. 2006). It is a valid and reliable measure recording the time it takes for a participant to perform five transfers from sitting to standing from a chair without using their arms (Smeets et al. 2006; Tsunoda et al. 2013). This test was included as an objective physical performance measure to assess the differences between the two groups.

#### Pedometers

Pedometers have been used as a measure of physical activity in a number of studies (Crouter et al. 2003; Lam et al. 2012; Mestek et al. 2008). It is acknowledged that some literature reports pedometry as not being a valid measure of physical activity overall (Crouter et al. 2003). However, in the present study, pedometers were used to compare levels of activity between groups rather than as absolute measures of physical activity. As pedometers are inexpensive and easy to use with acceptable reliability, they provide adequate data to allow for comparison between groups. Oregon Scientific^®^ PE326PM Pedometers were used in this study.

#### Body mass index

Body mass index (BMI) is a commonly used measure that aims to determine body fat in adults and adolescents and has been found to be both reliable and valid (Garrouste-Orgeas et al. 2004). In addition, Pietrobelli and colleagues found the BMI to be a reliable screening tool when used on a group of people (Pietrobelli et al. 1998). BMI can be expected to reflect levels of physical activity with those who are less physically active having higher BMI.

### Procedure

Ethical approval was obtained from the University of Cape Town, Faculty of Health Sciences Human Research Ethics Committee (HREC REF 093/2015) and the Groote Schuur Hospital Human Research Ethics Committee. Once ethical approval was granted, people with chronic pain were identified for the chronic pain group from the waiting list at the CPMC at GSH and were contacted via telephone call and screened. Volunteers for the healthy matched controls were identified from the communities of those with chronic pain and were telephoned and screened as described above.

For both groups, initial confirmation of willingness and consent to participate in the study was obtained verbally via telephone prior to written informed consent being obtained on visiting GSH for data collection. The researchers underwent training in the administration of the outcome measures including the questionnaires and objective measures of physical activity.

All those who met the inclusion criteria were invited to attend an appointment at the GSH outpatient physiotherapy department where the study objective was explained to them and written informed consent was obtained. The BPI and IPAQ were administered by interview. The responses to the BPI were used to categorise participants as being in the pain group or no-pain group. Responses to the IPAQ were used to group the participants into low, moderate or high physical activity levels. The 6MWT and the RSST were performed with standardised instructions according to an instruction sheet. The 6MWT was performed using a standardised track and distance (20 m shuttles) and the RSST was performed using a standardised chair. These tests were included as objective physical performance measures to assess the differences between the two groups. The participants’ weight and height were measured using standardised instruments to calculate BMI as kg/m^2^. On completion of testing, participants were provided with pedometers and instructed in their use. Following pedometer demonstration, participants were requested to demonstrate their use to the researchers. Participants wore the pedometers for a period of one week (seven consecutive days) for all waking hours. Participants were contacted daily by telephone to record measures, ensure correct use of the equipment and monitor for technical complications. All data were recorded on an excel spreadsheet for statistical analyses.

### Ethical consideration

The Declaration of Helsinki (World Medical Association 2013) was adhered to throughout the duration of the study, following the principles of justice, autonomy, beneficence and non-maleficence (Finch, Geddes & Larin 2005). Participants were educated prior to recruitment to the study and given the opportunity to withdraw at any stage which would not affect their position on the waiting list for treatment at the CPMC, ensuring autonomy. Beneficence was ensured with participants educated on chronic pain and the benefits of physical activity. Non-maleficence was ensured with assessment tools having minimal risks.

The cost of transport was reimbursed for all participants. Confidentiality was maintained throughout the study by the use of coding. The information has only been used for the purposes of this study and anonymity was ensured as participants’ names and personal details were not disclosed to anyone other than the researchers.

### Statistical analyses

As only 24 participants were recruited to the study with 12 participants in each group, non-parametric analysis was conducted. Differences between groups were tested using the Mann–Whitney *U* test. Data were analysed using Statistica software (StatSoft 2007). Results are presented as median (range) with significance accepted as *p* < 0.05.

## Results

The socio-demographic characteristics of the participants will be presented prior to presentation of physical activity levels for those with chronic pain and the matched controls.

There were eight females and four males in each group. As shown in Table 1, there were no significant differences between groups for age and height. However, there was a significant difference in BMI with the BMI for the chronic pain group being significantly higher than that of the control group (29.36 kg/m^2^ [18.94–34.63] vs 22.16 kg/m^2^ [17.1–30.86]; *U* = 34; *p* < 0.03).

The majority of participants in both groups had occupations which were predominantly sitting (45.83%) showing a sedentary lifestyle (Table 2). There was no significant difference between groups for occupational characteristics (χ^2^ = 6.88; *p* = 0.14).

As expected, the chronic pain group had significantly worse scores on the BPI compared to the control group for the PSS (6.75 [4–9.5] vs 0.75 [0–2.25]; *U* = 0; *p* < 0.01) as well as the PIS (7.57 [5.14–9.28] vs 0 [0–0.57]; *U* = 0; *p* < 0.01).

**TABLE 1:** Physical characteristics of participants (*N* = 24).

Characteristic	Chronic pain (*n* = 12)	Matched control (*n* = 12)	Statistical test
Age (years)	43 (19–54)	47 (20–56)	*U* = 67; *p* = 0.79
Height (m)	1.65 (1.55–1.83)	1.68 (1.5–1.89)	*U* = 51.5; *p* = 0.25
Weight (kg)	81 (52.2–105)	63.5 (46–90)	*U* = 44.5; *p* = 0.12
BMI (kg/m^2^)	29.36 (18.94–34.63)	22.16 (17.1–30.86)	*U* = 34; *p* = 0.03

**TABLE 2:** Occupational characteristics of all participants (*N* = 24).

Occupation	All participants (*n* = 24)	Chronic pain (*n* = 12)	Matched control (*n* = 12)
Standing/sitting	4	3	1
Sitting	11	3	8
Standing	2	1	1
Unemployed	7	5	2

**TABLE 3:** Frequency table for levels of physical activity on the IPAQ (*N* = 24).

Level of physical activity	All participants (*n* = 24)	Chronic pain (*n* = 12)	Matched controls (*n* = 12)
Inactive	14	6	8
Minimally active	9	6	3
Health-enhancing physical activity	1	0	1

**FIGURE 1:**
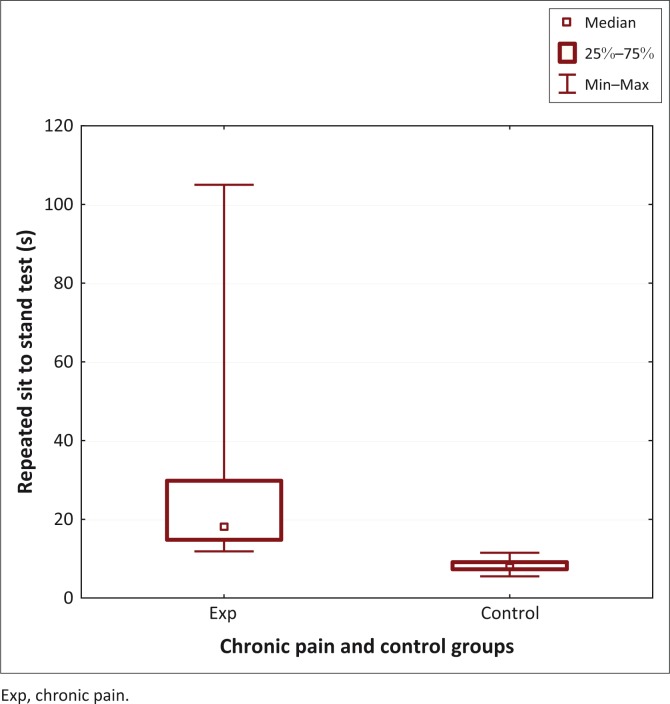
Time taken for repeated sit-to-stand test for the chronic pain and control groups.

### Measures of physical activity

There was no significant difference between the groups for the IPAQ scores in any of the physical activity categories (Table 3). Both groups had low levels of physical activity with 58.33% (14) of the participants classified as being inactive.

**FIGURE 2:**
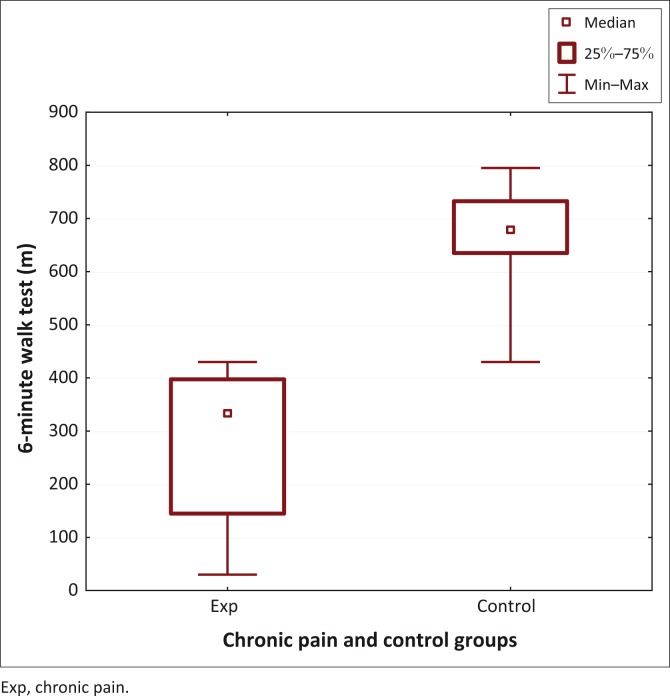
Distance walked in 6-minute walk test for the Chronic Pain and Control Groups.

Objective measures of physical activity showed significant differences between the two groups. The chronic pain group had significantly worse scores for the RSST (17.9 s [11.83–105] vs 7.85 s [5.5–11.5]; *U* = 0; *p* < 0.01) (Figure 1) and the 6MWT (335 m [30–430] vs 680 m [430–795]; *U* = 0.5; *p* < 0.01) (Figure 2).

**FIGURE 3:**
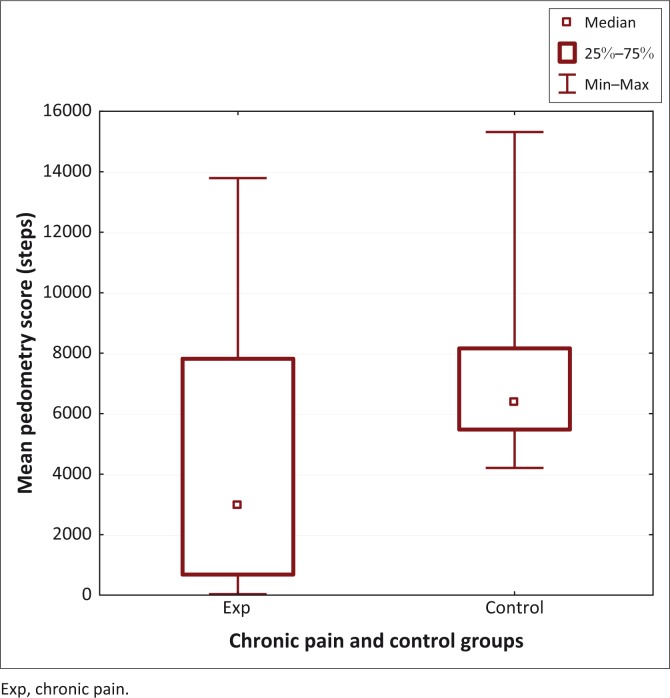
Mean pedometry scores for 7 days for the chronic pain and control groups.

The chronic pain group had significantly lower scores for the mean daily pedometry readings (2985.1 [32.8–13785.4] vs 6409.4 [4207.1–15313.6]; *U* = 35; *p* < 0.03) (Figure 3) and the total pedometry readings (20 896 [229–96 526] vs 44865.5 [29 450–107 195]; *U* = 35; *p* < 0.03).

## Discussion

The aim of this study was to determine the levels of physical activity in people with chronic pain compared to healthy individuals, specifically in South Africa as there is a paucity of data in this regard. The main findings were that people with chronic pain had significantly lower levels of physical activity compared to healthy individuals with all participants having low levels of physical activity. The participants with chronic pain performed significantly worse on physical performance tests (RSST and 6MWT), showing a reduced capacity for physical activity. This result is as expected and is in line with previous studies (Griffin, Harmon & Kennedy 2012; Ryan et al. 2009).

The Fear Avoidance Model (Leeuw et al. 2007) hypothesises that people with chronic pain avoid physical activity with the belief that physical activity will aggravate their pain. These people have an altered movement pattern as they move sporadically, in a fear-motivated attempt to avoid inducing a painful episode. This fear avoidance behaviour contributes to decreased levels of physical activity and contributes to increasing levels of disability in people with chronic pain (Ryan et al. 2009; Verbunt et al. 2001). Changes in movement patterns cannot be inferred from pedometry results as the pedometers calculate a mean daily reading. However, changes in movement pattern are clearly demonstrated by the RSST and 6MWT. When the participants with chronic pain were put under the pressure of a physical performance test, they did not have the tolerance to raise their levels of performance.

The pedometry findings showed that those with chronic pain had significantly lower scores than the matched controls. However, the presence of two outliers in the chronic pain group requires consideration (Figure 3). These two participants with chronic pain did more physical activity than many of the matched controls. This demonstrates the Avoidance-Endurance Model (Plaas et al. 2014). The Avoidance-Endurance model proposes that while the majority of people with chronic pain avoid physical activity, a subgroup ignore their pain and push themselves physically despite their pain. This model describes the maladaptive behaviour of people with chronic pain with the people who avoid physical activity becoming disabled as a result of immobility and muscle atrophy with further development of chronic pain because of neurophysiological processes of sensitisation, while those who endure their pain may not become disabled but develop further pain as a consequence of muscle and joint overuse leading to sensitisation.

The mean BMI of the chronic pain group fell into the classification of obese. The literature reports that people with low levels of physical activity have an increased BMI (Blair & Church 2004), suggesting that those in the present study with higher BMIs may have lower levels of activity. This result could infer that the cycle of inactivity in people with chronic pain perpetuates and contributes to comorbidities leading to a greater burden of disease (Van Hecke, Torrance & Smith 2013). The South African population as a whole has been found to have relatively low physical activity levels (Joubert et al. 2007), placing a large burden on health care services through an increase in non-communicable chronic diseases and conditions, increased disability and increases in chronic pain (Strydom 2013). With this growing burden in South Africa, a focus on levels of physical activity in people with chronic health conditions is indicated.

As confirmed in this study and as previously described elsewhere (Guthold et al. 2008; Joubert et al. 2007), the general population of South Africa appears to have low levels of physical activity. It appears that people with chronic pain have an even lower level of physical activity compared to the general population, shown in this study by poor performance on physical tests (RSST and 6MWT) and pedometry. These results, therefore, indicate that it is important to ensure that chronic pain management includes a focus on physical activity to reduce the burden of disease. There is a specific role for physiotherapy in this management as physiotherapists are experts in exercise prescription and can prescribe graded exercises for people with chronic pain as the primary treatment (Hauser et al. 2010). In combination with graded exercise, physiotherapy treatment should include education on pain neurophysiology to facilitate patient’s shifting of fear avoidance beliefs (Louw et al. 2011). Physiotherapy targeting an increase in physical activity in people with chronic pain aims to break the cycle of inactivity which contributes to an increase in BMI and increased risk of developing comorbidities with an associated decrease in quality of life.

### Study limitations

Because of the restricted availability of participants from the CPMC, limited finances and limited time, the intended sample size of this study was not reached. This limits the generalisability of the results. However, the results showed a significant difference in the primary outcome measure of pedometry and for the 6MWT and RSST between those with pain and those without pain. This significant difference, even in a small sample, suggests that there is indeed a difference in levels of physical activity between those with chronic pain and healthy controls.

The reliability of the IPAQ and pedometry is limited and could have been improved with a larger sample size. Further, more accurate data could have been obtained through the use of accelerometers which record more detail about movement patterns than pedometers which only record steps taken. The use of pedometers may have limited the validity of the measure of physical activity. In addition, as this was a cross-sectional study, it was not possible to identify a cause and effect relation between chronic pain and physical activity which limits the impact of the study. Therefore, future longitudinal studies with larger samples are indicated to identify causal relationships. Further limitations of the study were that differences in physical activity levels between males and females in people with chronic pain as well as differences in physical levels in different age groups were not established and could benefit from further research.

The use of the BMI was potentially limiting in its depth by not assessing the influence of the participants’ perception of BMI. Future studies should include perception of BMI in their design. In addition, the controls were matched to the members of the pain group for all variables but weight; this could influence the difference in physical activity levels and is a weak point of the study.

## Conclusion

Participants with chronic pain had a reduced capacity for physical activity. The pedometry results illustrate a range of maladaptive strategies adopted by those with chronic pain. The majority of people with chronic pain appear to avoid physical activity leading to greater disability as a result of immobility and muscle atrophy. However, a small subgroup appears to ignore their pain and push themselves physically despite their pain. This perseverance behaviour leads to further pain as a consequence of muscle and joint overuse. Both maladaptive behavioural responses result in further sensitisation of the central nervous system. The method used to target physical activity in these patients should be considered in treatment planning, specifically for physiotherapy. As the South African population has low levels of physical activity compared to global levels, it is crucial to address this issue to reduce the burden of disease. Future treatment of chronic pain by physiotherapists should focus on graded exercise prescription and education on pain neurophysiology with the aim of improving the individual’s levels of physical activity in daily living thereby reducing disability.
